# How to assess response

**DOI:** 10.1007/s00406-024-01834-8

**Published:** 2024-06-05

**Authors:** Steffen Zitzmann, Christoph Lindner

**Affiliations:** 1 Medical School Hamburg, Hamburg, Germany; 2 Max Planck Institute of Psychiatry, Munich, Germany

The term response has been established as a key concept in psychiatry. It is an indispensable part of the jargon and frequently applied in practice and research. By a patient who showed a response, we mean someone whose symptoms improved to an extent that is practically important. Often, it is implied that this improvement has occurred in response to how the patient was treated, such as by taking a drug. In clinical research, however, the term response often refers to the improvement alone without a clear reference to the specific cause for this improvement. The reason is that we can hardly know how this patient would have improved if they had not been treated or had received a placebo.

To assess whether a patient has responded, their percentage symptom reduction from baseline has to be obtained first. This value can be computed as $\left({S}_{2}/{S}_{1}-1\right)\cdot 100\text{\%}$, where ${S}_{1}$ and ${S}_{2}$ stand for the patient's symptoms at baseline and after the treatment, respectively [1]. Then, the patient can be classified into a category of improvement. These categories are based on cutoffs, for example, on steps of $25\text{\%}$ [2]. However, there is an issue with this procedure, which arises because interviews and questionnaires are often used. Such measures are blurred by noise called measurement error [3, 4]. The amount of error is typically expressed by the measure’s reliability, which is defined as the proportion of the variability of the measured score that can be attributed to true differences between patients [5].

To illustrate, consider the Positive and Negative Syndrome Scale (PANSS) [6]—a measure of symptom severity in schizophrenia. Recent research found that the internal consistency of the PANSS (i.e., an estimate of its reliability) ranged up to 0.94 [7]. Before we continue, note that to compute a patient’s percentage PANSS reduction from baseline, their total scores need to be corrected by subtracting 30 points [9, 10]. Now, suppose the patient got a corrected total score of ${S}_{1}^{*}=60$ points at baseline and ${S}_{2}^{*}=40$ after treatment. Using the equation for computing percentage symptom reduction from baseline, their symptoms seemingly decreased by $33.3\text{\%}$. Thus, if measurement error was ignored, the patient would be classified into the 25 to $50\text{\%}$ improvement category, meaning the patient would be described as having improved. However, we may indicate uncertainty due to measurement error by a confidence interval. The interval can be computed as:

$$\left({S}_{2}^{*}/{S}_{1}^{*}-1\right)\cdot 100\text{\%}\pm 1.96\cdot \sqrt{\left(1-\rho \right)\cdot \left(1+{S}_{2}^{*2}/{S}_{1}^{*2}\cdot {\sigma }_{1}^{2}/{\sigma }_{2}^{2}\right)\cdot {\sigma }_{2}^{2}/{S}_{1}^{2}}\cdot 100\text{\%}$$

where $\rho$ is the reliability of the PANSS. ${\sigma }_{1}$ and ${\sigma }_{2}$ are the standard deviations at baseline and after treatment [1, 11]. Consider the patient whose symptoms reduced from 60 to 40 points. If, for example, the reliability of $\rho =.94$ is used in the calculation of the patient’s confidence interval, and the same standard deviation of ${\sigma }_{1}\left(={\sigma }_{2}\right)=10.93$ is assumed at baseline and after treatment, we yield $-33.3\text{\%}\pm 10.5\text{\%}$. Thus, we cannot be certain that their actual improvement was not $23\text{\%}$ instead of $33.3\text{\%}$, for example. More importantly, this interval includes $25\text{\%}$, indicating that this patient could fall into the 0 to $25\text{\%}$ category (i.e., no practically important improvement). In other words, it cannot be decided whether this patient belongs to the 0 to $25\text{\%}$ or 25 to $50\text{\%}$ category.

**Fig. 1:**
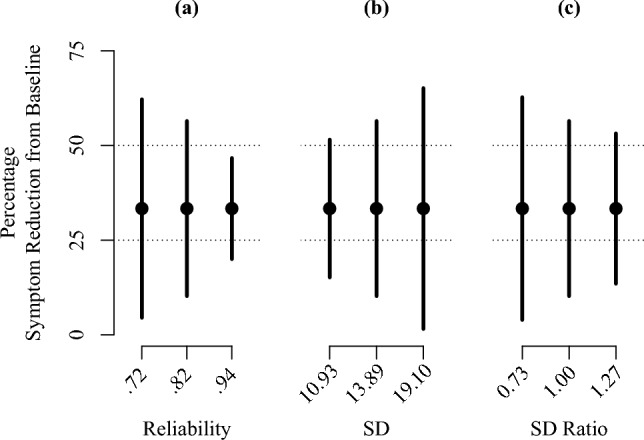
Dependence of the confidence interval on reliability $\uprho$ [Panel (a)], standard deviation ${\upsigma }_{1}\left(={\upsigma }_{2}\right)$ [Panel (b)], and standard deviation ratio ${\upsigma }_{1}/{\upsigma }_{2}$ [Panel (c)]. *SD* standard deviation

It should be noted that the confidence interval involves making assumptions about the measure, particularly regarding quality and distributional aspects. Thus, depending on the context, one may use different values for the reliability and the standard deviations in the calculation. To illustrate how they impact the patient’s confidence interval, see Fig. 1. Panel (a) shows that when the level of reliability $\rho$ is increased, the interval will be narrowed. By contrast, increasing the standard deviation ${\sigma }_{1}\left(={\sigma }_{2}\right)$ will increase the width [Panel (b)]. Finally, given unequal standard deviations, a greater ratio ${\sigma }_{1}/{\sigma }_{2}$ will lead to a decrease in width [Panel (c)].

To conclude, the patient whose confidence interval includes a cutoff illustrates the need for a refinement of the assessment of response. Interviews and questionnaires are not error-free measures. As psychiatrists, we should take this message serious and “embrace” the uncertainty. To decide whether a patient has responded, we should not focus solely on their percentage symptom reduction from baseline. In addition, a confidence interval should be placed around this value so that it can be better evaluated into which improvement category the patient falls. If the interval includes a cutoff, a definite categorization cannot be made, and further information has to be gathered.
